# Impact of intensive care unit supportive care on the physiology of Ebola virus disease in a universally lethal non-human primate model

**DOI:** 10.1186/s40635-019-0268-8

**Published:** 2019-09-13

**Authors:** Guillaume Poliquin, Duane Funk, Shane Jones, Kaylie Tran, Charlene Ranadheera, Mable Hagan, Kevin Tierney, Allen Grolla, Amrinder Dhaliwal, Alexander Bello, Anders Leung, Cory Nakamura, Darwyn Kobasa, Darryl Falzarano, Lauren Garnett, Hugues Fausther Bovendo, Heinz Feldmann, Murray Kesselman, Gregory Hansen, Jason Gren, George Risi, Mia Biondi, Todd Mortimer, Trina Racine, Yvon Deschambault, Sam Aminian, Jocelyn Edmonds, Ray Sourette, Mark Allan, Lauren Rondeau, Sharron Hadder, Christy Press, Christine DeGraff, Stephanie Kucas, Bradley W. M. Cook, B. J. Hancock, Anand Kumar, Reeni Soni, Darryl Schantz, Jarrid McKitrick, Bryce Warner, Bryan D. Griffin, Xiangguo Qiu, Gary P. Kobinger, Dave Safronetz, Derek Stein, Todd Cutts, James Kenny, Geoff Soule, Robert Kozak, Steven Theriault, Liam Menec, Robert Vendramelli, Sean Higgins, Guodong Liu, Niaz Md Rahim, Samantha Kasloff, Angela Sloan, Shihua He, Nikesh Tailor, Michael Gray, James E. Strong

**Affiliations:** 1 National Microbiology Laboratory, Public Health Agency of Canada, 1015 rue Arlington Street, Winnipeg, Manitoba R3E 3R2 Canada; 2 Department of Pediatrics & Child Health, College of Medicine, Faculty of Health Sciences, University of Manitoba, Winnipeg, Manitoba Canada; 3 Department of Infectious Diseases and Medical Microbiology, College of Medicine, Faculty of Health Sciences, University of Manitoba, Winnipeg, Manitoba Canada; 4 Department of Anaesthesia and Medicine, College of Medicine, Faculty of Health Sciences, University of Manitoba, Winnipeg, Manitoba Canada; 5 Medtronic Canada, Winnipeg, Manitoba Canada; 6 National Centre for Foreign Animal Disease, Canadian Food Inspection Agency, Winnipeg, Manitoba Canada; 7 Vaccine and Infectious Disease Organization-International Vaccine Centre, University of Saskatchewan, Saskatoon, Canada; 8 Centre de Recherche en Infectiologie, Centre Hospitalier Universitaire de Québec, Université Laval, Québec, Canada; 9 Laboratory of Virology, Division of Intramural Research, National Institute of Allergy and Infectious Diseases, National Institutes of Health, Hamilton, USA; 10 Faculty of Critical Care, Royal University Hospital, Saskatoon, Saskatchewan Canada; 11 Infectious Disease Specialists, P.C., Missoula, MT USA; 12 Arthur Labatt Family School of Nursing, Western University, London, Ontario Canada; 13 Child & Women’s Health Programme, Winnipeg Regional Health Authority, Winnipeg, Manitoba Canada; 14 Cytophage Technologies, Inc., St. Boniface Hospital, Albrechtsen Research Centre, Winnipeg, Manitoba Canada; 15 Department of Surgery, Division of Pediatric Surgery, College of Medicine, Faculty of Health Sciences, University of Manitoba, Winnipeg, Manitoba Canada; 16 Regional Pharmacy, Winnipeg Regional Health Authority, Winnipeg, Manitoba Canada; 17 Department of Laboratory Medicine & Pathobiology, University of Toronto, Toronto, Ontario Canada

**Keywords:** Ebola, Supportive care, Fluid, NHP, Vasoactives, Hydrocortisone, Ventilatory support, Pathophysiology

## Abstract

**Background:**

There are currently limited data for the use of specific antiviral therapies for the treatment of Ebola virus disease (EVD). While there is anecdotal evidence that supportive care may be effective, there is a paucity of direct experimental data to demonstrate a role for supportive care in EVD. We studied the impact of ICU-level supportive care interventions including fluid resuscitation, vasoactive medications, blood transfusion, hydrocortisone, and ventilator support on the pathophysiology of EVD in rhesus macaques infected with a universally lethal dose of Ebola virus strain Makona C07.

**Methods:**

Four NHPs were infected with a universally lethal dose Ebola virus strain Makona, in accordance with the gold standard lethal Ebola NHP challenge model. Following infection, the following therapeutic interventions were employed: continuous bedside supportive care, ventilator support, judicious fluid resuscitation, vasoactive medications, blood transfusion, and hydrocortisone as needed to treat cardiovascular compromise. A range of physiological parameters were continuously monitored to gage any response to the interventions.

**Results:**

All four NHPs developed EVD and demonstrated a similar clinical course. All animals reached a terminal endpoint, which occurred at an average time of 166.5 ± 14.8 h post-infection. Fluid administration may have temporarily blunted a rise in lactate, but the effect was short lived. Vasoactive medications resulted in short-lived improvements in mean arterial pressure. Blood transfusion and hydrocortisone did not appear to have a significant positive impact on the course of the disease.

**Conclusions:**

The model employed for this study is reflective of an intramuscular infection in humans (e.g., needle stick) and is highly lethal to NHPs. Using this model, we found that the animals developed progressive severe organ dysfunction and profound shock preceding death. While the overall impact of supportive care on the observed pathophysiology was limited, we did observe some time-dependent positive responses. Since this model is highly lethal, it does not reflect the full spectrum of human EVD. Our findings support the need for continued development of animal models that replicate the spectrum of human disease as well as ongoing development of anti-Ebola therapies to complement supportive care.

**Electronic supplementary material:**

The online version of this article (10.1186/s40635-019-0268-8) contains supplementary material, which is available to authorized users.

## Background

The 2013–2016 West Africa Ebola virus disease (EVD) outbreak was the largest in recorded history [1], and despite decades of research, no approved treatment for Ebola was available. Consequently, supportive care was the only viable option outside of clinical trials. This remains the case in the current 2018 Democratic Republic of Congo outbreak, now the second largest ever recorded [2]. There have been some anecdotal observations to suggest supportive care is effective in caring for patients infected with filoviruses. The striking difference in mortality rate between the 1967 Marburg virus outbreak in Germany and Yugoslavia (20–25%) compared to subsequent outbreaks in Africa (70–85%) has been attributed to higher-quality medical care in Europe [3]. During the Kikwit EVD outbreak, mortality decreased for the last 25 patients (from 79 to 56%) [4] following the arrival of Médecins Sans Frontières and institution of limited supportive care [5]. The significance of the decreased mortality has been questioned in light of the small sample size, the uncertain impact of convalescent plasma (given to 8/25 patients), and the 100% mortality experienced by 6 patients who received parenteral fluid earlier on in the outbreak [4].

The combination of anecdotal evidence and perceived similarities between EVD and sepsis pathophysiology has translated into a belief that supportive care alone will provide a survival advantage to EVD patients [3, 5, 6]. The EVD-specific evidence for this is limited; for example, fluid replacement recommendations rely primarily on evidence from other diseases (e.g., cholera) [5]. Furthermore, the provision of supportive care is logistically difficult within Ebola treatment centers, a problem compounded by the safety considerations of basic acts such as intravenous (IV) starts when the potential consequences of needlestick injuries are considered. Since the impact of supportive care alone on the pathophysiology of EVD is presently unknown, there is an urgent need to study its role in EVD patients. Such studies are difficult to perform in humans due to the conditions within outbreak settings [7]. As a result, experimental evidence of the impact of specific components of supportive care on survival can be gleaned using high-quality animal models.

We studied the impact of ICU-level supportive care interventions including fluid resuscitation, vasoactive medications, blood transfusion, hydrocortisone, and ventilator support on the pathophysiology of EVD in rhesus macaques infected with a universally lethal dose of Ebola virus strain Makona C07.

## Methods

We previously described our model for non-human primate (NHP) intensive care [8]. In brief, rhesus macaques were managed continuously by bedside teams and study physicians in a biosafety level 4 (BSL4) environment. The NHPs were sedated throughout the experiments. We used endotracheal intubation and a pressure-regulated volume control strategy with spontaneous ventilation to maintain oxygenation/ventilation. Intravenous access was established with two triple lumen central venous catheters. Two arterial lines were placed for continuous invasive blood pressure (BP) monitoring. Other vital signs including temperature, end-tidal CO_2_, non-invasive heart rate (HR), respiratory rate, and oxygen saturation were continuously monitored. Blood sampling was performed preferentially through arterial lines, but venous lines were used if needed. Intubation/ventilation, line placement, and stabilization were attained prior to infection.

Initial fluid balance was closely monitored to establish and then maintain euvolemia, with a goal of maintaining balance between inputs (fluids for line patency, maintenance intravenous fluid, medications, and feeds) and outputs (urine, vomiting, nasogastric aspirates, and water loss stools) as well as insensible losses (calculated at 400 mL/m^2^/day). Sampled blood volumes were deemed to be insignificant to overall fluid balance and thus not included as an output. Fluid goals included a minimal urine output of 1 mL/kg/h [9] with a goal to maintain an overall running total fluid balance of ± 300 mL, tabulated every 12 h.

All four sedated animals were observed following intubation and line access to ensure equipment function, and cardiorespiratory stability. The observation period was variable (23–116 h), with the 116-h delay animal representing an outlier that developed a ventilator-associated pneumonia (VAP) prior to infection. Once deemed stable, the NHPs were infected with a pre-planned dose of 1000 plaque-forming units of Ebola virus strain Makona C07 via two separate intramuscular injections, one in each thigh. This approach is the standard used in Ebola challenge experiments and results in a universally lethal model [10]. The virus preparation was then back titrated by tissue culture infectious dose 50% (TCID_50_) to confirm delivered dose [11]. Additionally, a fifth animal (serving as pilot and control) had been maintained under similar study conditions but left uninfected.

Pre-determined interventions for the acute phase of illness included the use of isotonic crystalloid boluses of 10 or 20 mL/kg for blood pressure (BP) support targeting mean arterial pressure (MAP) of > 65 mmHg (human target value [12] in the absence of a rhesus-specific target), or signs of compensated shock (increasing heart rate and/or widening pulse pressure), followed by vasoactive medication support with norepinephrine and/or vasopressin if the animal was fluid unresponsive following 60 mL/kg. For analysis purposes, vasoactive medication doses were converted to the vasoactive-inotropic score (VIS) using the following validated formula [13]: epinephrine (dose in μg/kg/min × 100) + norepinephrine (dose in μg/kg/min × 100) + vasopressin (dose in U/kg/min × 10,000). Since multiple vasoactive medications were used, at times concurrently, conversion to a unified VIS enabled direct comparison of the relative potency of regimens in use over time.

Additionally, autologous whole blood transfusion, hydrocortisone, and antibiotics were available. We did not have pre-specified target values for these latter interventions, and treatment decisions were made by consensus of the in-house treating physicians. The study protocol was reviewed and approved by the institutional Animal Care Committee, in accordance with the Canadian Council on Animal Care (CCAC) guidelines [14].

## Results

### Overall clinical course

**Fig. 1:**
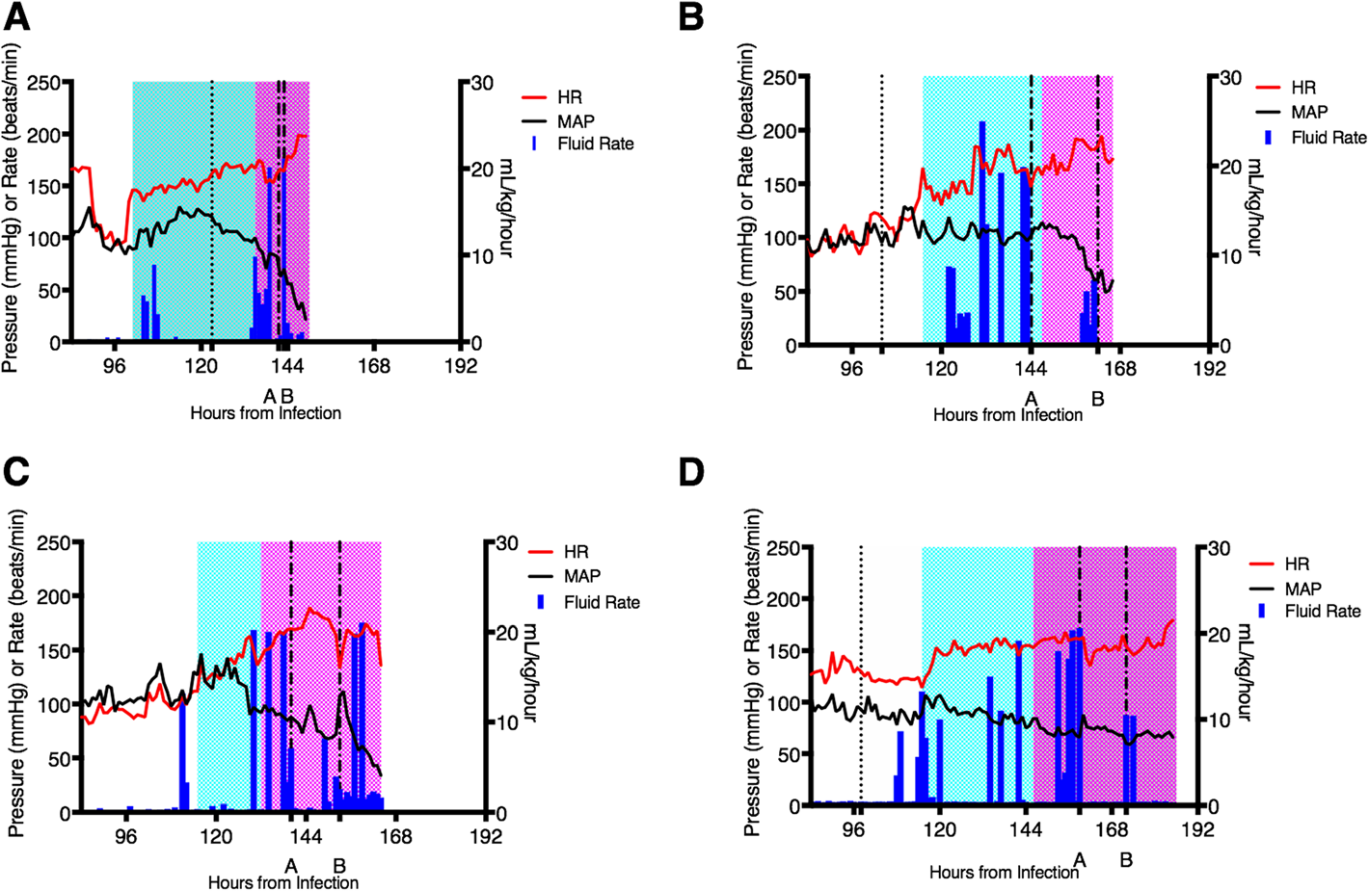
Cardiovascular vital sign trends and fluid Resuscitation. Trends in cardiovascular vital signs in relation to interventions. **a**–**d** NHP1–4, respectively. HR (measured in beats per minute) and MAP (measured in mmHg) are plotted on the left *Y*-axis. The vertical dashed line in **a**, **b**, and **d** represents the first detection of viremia (for NHP3 (**c**), first detection occurred earlier than this time scale). The vertical lines labeled “A” and “B” in those same panels represent the start of the first and second vasoactive medications, respectively. Cyan-shaded areas represent compensated shock while magenta-shaded areas represent decompensating shock. The vertical blue bars denote fluid rate in mL/kg/hour; bars significantly above the baseline represent fluid boluses. The initial high heart rate for NHP1 (**a**) is secondary to acidosis. Once the acidosis began to resolve, the heart rate stabilized until the onset of EVD-related illness

Despite being outbred animals, all four NHPs demonstrated very similar cardiovascular patterns (Fig. 1). Table 1 provides a comparison of critical time points in the infected animals’ clinical courses. Overall, animals 2–4 (NHP2, NHP3, and NHP4) demonstrated remarkably similar clinical courses. The first infected animal (NHP1) had a more complex course secondary to a ventilator-associated pneumonia (VAP) as well as a renal tubular acidosis unrelated to the EBOV infection [8]. These complications delayed time to infection compared to NHP2–4 to allow for treatment of the VAP with antibiotics prior to infection. The acidosis was diagnosed after infection but was corrected prior to the onset of EVD-related signs (Fig. 1). Due to the variable time to infection, we present data from 20 h pre-infection to the terminal endpoint to facilitate comparison.

**Table 1:** Important experimental time points

Time point	NHP1	NHP2	NHP3	NHP4	Average	Standard deviation
Onset of compensated shock	101	115	115	116	111.8	7.2
Onset of decompensated shock	135	147	132	146	140	7.6
Start of fluid resuscitation	135	131	130	134	132.5	2.4
Start of the first vasoactive agent	141.5	144	140	159	146.1	8.7
Average urine output (mL/kg/h)	4.7	5.5	4.9	4.3	4.9	0.5
Start of the second vasoactive agent	143	162	153	172	157.5	12.4
Blood transfusion	145	149	148	145	146.8	2.1
Onset of fever	123	102	Remained afebrile	121	115.3	11.6
Onset of viremia	120*	104	80	100	101	16.5
Time to terminal endpoint	150	166	164	186	166.5	14.8

*Viral load was performed once daily for this animal, leading to probably late detection; see Fig. 3

The first vital sign to noticeably change was a rising heart rate (HR), beginning on average at 111.8 ± 7.2 h post-infection (HPI) (range 101–116). This is labeled as the start of compensated shock (cyan area, Fig. 1). A sustained decrease in mean arterial pressure (MAP) heralded the start of decompensated shock (magenta area, Fig. 1), starting on average 145 ± 7.2 HPI (range 135–152). Timing of interventions was variable, and these are described further below. Time to terminal endpoint, defined as irreversible arrhythmia or non-perfusing blood pressure, averaged 6.9 days (166.5 ± 14.8 HPI). This is comparable to the survival range (6–8 days post-infection) observed in rhesus macaques using this same gold standard model but without the ICU-style supportive care (Additional file 1: Table S1) [15–17]. The control animal, in contrast, had a stable clinical course up to experimental hour 216, when it was euthanized upon study completion (Additional file 2: Figure S1).

Rhesus macaques have a higher baseline temperature than humans. As such, fever was defined as a sustained temperature above 39.5 °C [18]. Onset of fever was variable, with NHP1 developing a sustained fever starting at 123 HPI, while NHP2 developed a fever between 102 and 142 HPI, while NHP4 was febrile between 118 and 165 HPI. Both of these animals were afebrile for approximately 20 h prior to terminal endpoint. Interestingly, NHP3 remained afebrile throughout. Hourly urine output was well above our goal of 1 mL/kg/h (Table 1) and our fluid balance at the start of the compensated shock phase was also within target until the start of the compensated shock phase. Of note, anuric renal failure developed in both NHP2 and NHP4 (140 and 176 HPI), while NHP1 and NHP3 had ongoing, albeit reduced, urine output in late disease.

### Response to interventions

#### Fluid administration

All four animals received several boluses in response to tachycardia with or without associated hypotension starting between 130 and 135 HPI (Fig. 1). NHP1 received 71 mL/kg between 135 and 144 HPI. A combination of a more liberal approach and prolonged survival resulted in NHP2 receiving 145 mL/kg in boluses between 120 and 164 HPI, NHP3 receiving 138 mL/kg between 130 and 159 HPI, and NHP4 receiving 128 mL/kg. NHP1 additionally received 22 mL/kg between 104 and 108 HPI, although this corresponded to the peak of the renal tubular acidosis and may have been related to this phenomenon, rather than EVD.

While fluid boluses taken together did not have a consistently positive effect, we recognize that fluid responsiveness can change over time; thus, individual boluses were analyzed to look for fluid responsiveness over time. A 5% reduction in HR with a concomitant 5% rise in any BP parameter following the administration of fluid was determined to have been a successful bolus. Based on these criteria, only a proportion of boluses were deemed effective (NHP1, 1/6; NHP2, 3/8; NHP3, 2/7; and NHP4, 3/8). Importantly, all boluses deemed effective occurred early in the disease phase (137.1 ± 14.2 HPI).

#### Vasoactive medications

**Fig. 2:**
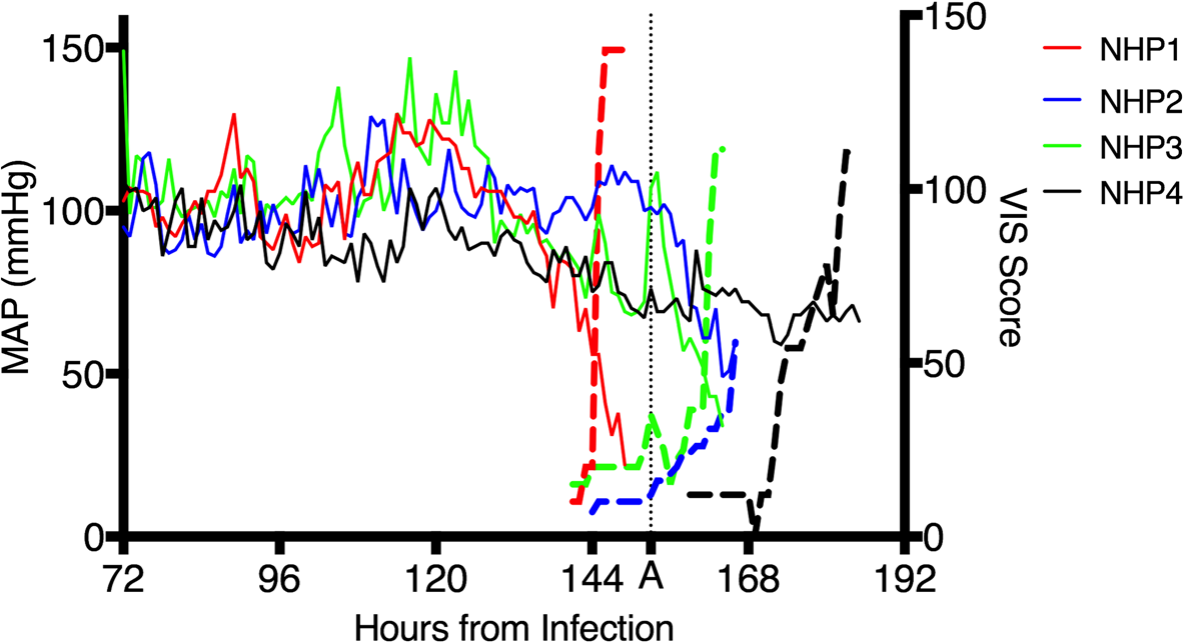
Effect of vasoactive medications on mean arterial pressure. Mean arterial pressure (solid colored lines plotted on the left *Y*-axis) compared to the VIS score (dashed lines plotted on the right *Y*-axis). The vertical dashed line labeled “A” denotes the start of vasopressin for NHP3. Note that the *X*-axis was truncated to focus on the late experimental stage for all four animals

The indication for starting vasoactive medications was loss of fluid responsiveness. The first vasoactive medication was introduced on average at 146.1 ± 8.7 HPI (range 140–159). There is a paucity of data with respect to optimal vasoactive medication choice in EVD, translating to iterative modification of approach during the experiments. Epinephrine was started first for NHP1 and NHP2, followed by norepinephrine. As seen in Table 1, NHP1 progressed to needing norepinephrine quickly while NHP2 had a longer delay. NHP3 differed by receiving norepinephrine first and vasopressin second. The use of vasopressin in NHP3 was prompted by substantial tachycardia that made the addition of norepinephrine less attractive. As seen in Fig. 2, vasopressin start is denoted by vertical line “A” and is followed by a substantial recovery in the MAP. While short-lived, this observation led to vasopressin being used as the initial vasoactive agent for NHP4 (see Fig. 2). Following conversion of the doses to the vasoactive-inotropic score (VIS), this was plotted against MAP (Fig. 2). Of note, the use of vasopressin first in NHP4 led to a stable MAP for approximately 13 h prior to the need for the start of norepinephrine. This is in contrast to the other regimens used, where continual dose escalation (as seen by the VIS) was needed to achieve the same effect.

#### Blood transfusion

All four animals received an autologous blood transfusion, occurring on average at 146.8 ± 2.1 HPI (range 145–149). In the absence of established NHP transfusion thresholds, the decision to transfuse was prompted by falling hemoglobin values potentially compromising oxygen delivery to the tissues, with the potential additional benefit of improving hemodynamic status, although evidence for this strategy may be lacking [19]. Following transfusion, hemoglobin rose for NHP1, NHP2, and NHP4 but fell for NHP3. The reason for the latter’s drop is uncertain, but this value was measured 5 h after the transfusion instead of within 1 h for the other animals. NHP3 may have progressed to a hemolytic process or fulminant DIC during the measurement delay. Notably, cardiovascular vital signs did not demonstrate substantial improvement in any animal. Lactate, as a surrogate of tissue oxygen delivery, rose in NHP1 and NHP4 but remained unchanged for NHP2 and NHP3.

#### Hydrocortisone

Hydrocortisone, at a dose of 2 mg/kg, was administered to all four animals. In the absence of a BSL4-compatible assay to measure cortisol, we used the clinical finding of fluid- and vasoactive-resistantshock as the trigger for administration. This led to substantial variation in time to first dose, ranging from 144 to 173 HPI. We did not observe a substantial effect of hydrocortisone on BP and HR, but the effects are often delayed and length of survival post-administration may not have been long enough to detect an effect.

#### Viremia and laboratory investigations

**Fig. 3:**
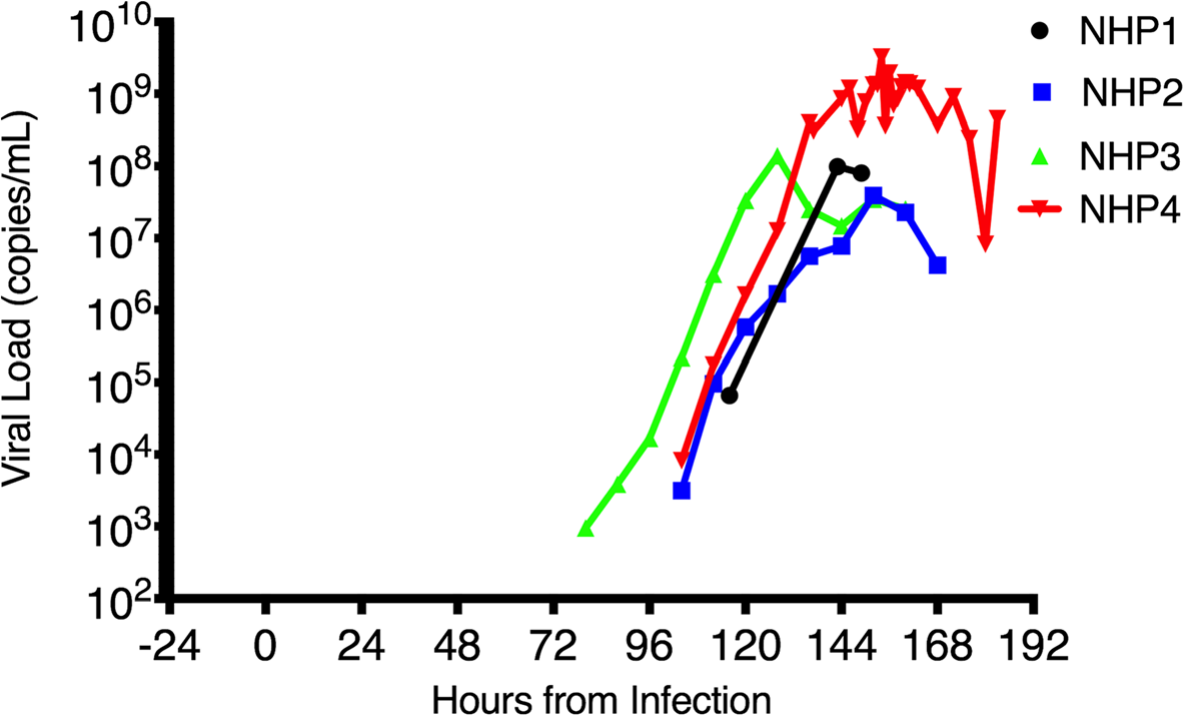
Viral load in plasma over time. Viral load, represented as genome copies/milliter of plasma, as derived from amplification of viral RNA using the National Microbiology Laboratory in-house assay run against a full EBOV plasmid as quantification standard

Viral load was measured in plasma and sampled every 24 h for NHP1, but this was found to be insensitive, so for NHP2–4, viral load was tested every 24 h for the first 3 days then every 8 h thereafter. NHP4 underwent sampling every 2 h between 86 and 102 HPI to better define early viral kinetics; protocol draws every 8 h resumed thereafter. Viral load over time is presented in Fig. 3. NHP2–4 became viremic within a 24-h window (80–104 HPI). NHP1 appears as a late outlier, but the viral load at first detection is substantially higher than the other three animals. This suggests delayed detection secondary to the less frequent sampling protocol for that experiment.

**Fig. 4:**
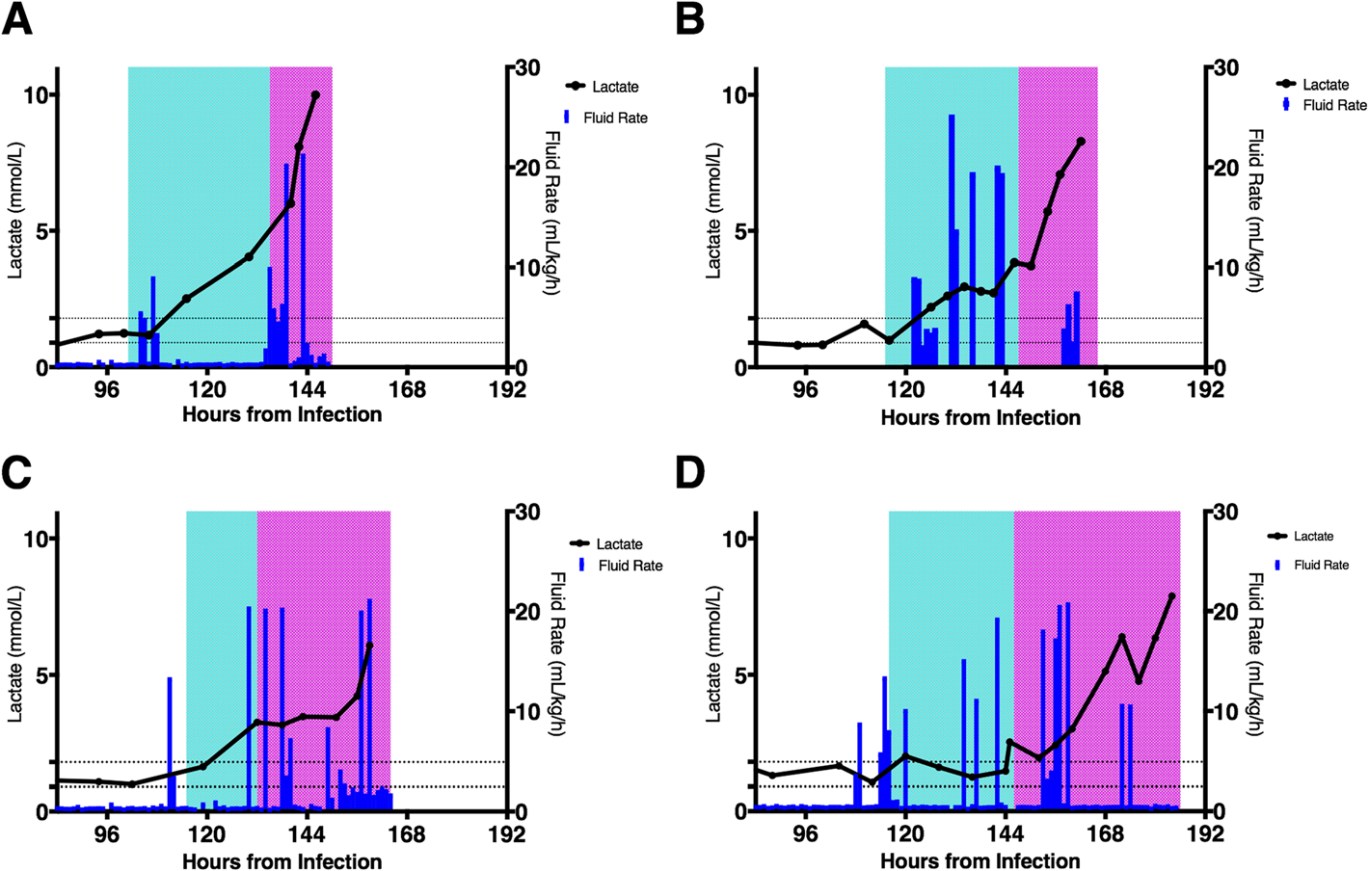
Lactate values over time. This figure trends lactate values over time for the four infected animals. **a**–**d** NHP1–4, respectively. **a**–**c** The lactate values over time (in black) for NHP1, NHP2, and NHP3, respectively. The blue columns represent bolus fluids. The horizontal dotted lines represent the normal value range. The cyan-shaded areas represent compensated shock while the magenta-shaded areas represent decompensating shock

Several biochemical parameters were measured throughout these experiments, but the most relevant was serum lactate. Lactate levels remained stable for the first several days and began to rise along with the onset of compensated shock (Fig. 4). A temporary plateau can be seen for NHP2–4, which correlates with the initial administration of fluid, followed by a resumption of the rise in spite of further fluids and additional interventions. NHP1 did not demonstrate a similar plateau, but the additional stressors of the ventilator-associated pneumonia (VAP) and acidosis may have negatively impacted the fluid response in this animal.

## Discussion

The impact of advanced organ-supporting care, in the form of fluid administration, vasoactive medication, blood transfusion, hydrocortisone, and continuous bedside care, resulted in limited and time-dependent effects on EVD pathophysiology. To our knowledge, this study represents the first experimental test of the effect of these interventions on the management of EVD in NHPs. This type of fundamental work is important since field evaluations of the impact of supportive care have thus far proven difficult. Notably, reduced mortality was observed in the 27 patients treated in developed health settings compared to West Africa (18.5% compared to 40%), but 25 of the 27 received at least 1 antiviral therapy, making the impact of supportive care difficult to discern from other therapies [20]. Additionally, a few centers were able to provide some elements of ICU-type care in West Africa, but the small number of patients treated and high variability in patient characteristics makes comparison of mortality rates at these centers with the broader outbreak context difficult [21, 22]. Thus, the work presented herein sheds important light on pathophysiology which cannot at present be gleaned by field observations.

There were several important observations during these experiments. The dynamic change in fluid responsiveness is notable, with early, but not late, fluid resuscitation improving both cardiovascular parameters (Fig. 1) and blunting the rise of lactate (Fig. 4). The lack of a sustained fluid response confirms an earlier finding that fluid administration alone in an EVD NHP model was ineffective [23]. Current EVD evidence-based guidelines focus heavily on fluid administration [3, 5, 6]. In field conditions, some EVD patients present for treatment in a fluid-depleted state following significant vomiting and diarrhea [24]. We did not test this particular scenario, since all four animals were euvolemic prior to the onset of shock, but we would expect that such patients would benefit from fluid replacement irrespective of EVD-related shock. The role of fluid replacement in EVD patients who have progressed to shock is unclear at this time, and our findings do not provide support for the efficacy of this strategy in isolation.

As with fluid administration, the addition of vasoactive medications did not have a sustained effect on maintaining the target MAP. There was variability regarding the combination of vasoactive medication used, but vasopressin appeared to have a better overall effect on supporting the MAP (Fig. 2). This may be related to the underlying pathophysiology (vasoplegic state) or related to the profound tachycardia observed in the animals, whereby additional adrenergic stimulation results in limited benefit. This is similar to the observation in humans of a lack of difference between vasopressin and norepinephrine in overall mortality but better performance of vasopressin in less severe septic shock [25]. Future studies focused on vasoactive medication choice may prove fruitful, both for optimal patient management but better delineation of underlying pathophysiology. Blood transfusions did not affect cardiovascular parameters but did result in an improvement in the hemoglobin value in three of the four animals. Hydrocortisone did not have an appreciable effect on cardiovascular dynamics, but filoviruses are known to affect the adrenal glands [26], so it remains possible that adrenal failure may play a role in a subset of patients and should be further evaluated.

The observations of temporary fluid and vasoactive medication responsiveness may shed light on a recent report that demonstrated that while generally ineffective during the West African outbreak, a subset of patients, with a moderate viral load, benefited from ICU-type supportive care [27]. We hypothesize that responsiveness to supportive care may enable patients with moderate disease to survive long enough for immune responses to control the infection. Those with mild disease may survive regardless, while those that progress rapidly to severe disease, as observed following intramuscular challenge, have a pathophysiology that is not amenable to supportive care interventions past a certain, as yet undefined, critical point. Future studies should focus on defining this critical event and identifying markers of moderate disease to enable appropriate triage. Furthermore, the existence of a subset of patients with disease that is untreatable with supportive care alone supports ongoing efforts to develop ebolavirus-specific countermeasures.

There are several important limitations to our findings. The most notable limitation was that only a small number of animals could be studied due to the continuous bedside care necessary to perform these studies. We are reassured by the consistent clinical progression amongst the animals, suggesting robust observations despite the small number of animals. While NHP1 experienced some iatrogenic complications, its clinical progression during the active disease phase was similar to the others. The continuous monitoring requirements also precluded the inclusion of an infected but un-intervened paired control animal. We did demonstrate that the model itself (e.g., ventilation, sedation) was not lethal through the uninfected pilot animal, which survived up to 10 days (Additional file 2: Figure S1).

Another limitation is the animal-to-animal protocol variability. These changes were instituted based on iterative analysis of the previous experiments. While this does make inter-animal comparisons more difficult, the ultimate finding (no impact on length of survival) was not affected by the protocol changes. Furthermore, given the ethical implications of NHP research, we felt it appropriate to ensure each experiment had an optimized chance of achieving the experimental outcome, rather than focus on having sufficient power to compare specific intervention types. The use of sedation medications may have had an impact on cardiovascular function, although we previously demonstrated that these medications were safe for long-term use (beyond the 10th day), exceeding the experimental window [8].

Finally, the route and dose of infection merits discussion. Natural human infection is thought to occur primarily through the mucosal route, resulting in an incubation period of approximately 9.5 days. By contrast, intramuscular infection is usually iatrogenic, resulting in a shorter incubation (6.3 days) as well as universal lethality [28]. Under ideal conditions, these experiments would have used a mucosal challenge to enhance external validity. Unfortunately, relatively little is known regarding mucosal infection in macaques and findings are inconsistent. A large inoculum (158,000 PFU) of the Mayinga strain resulted in lethal infections following oral or conjunctival challenge [29]. A small inoculum (10 PFU) of the Makona strain delivered to the oral or conjunctival surface failed to result in infection. A larger dose (100 PFU) orally proved lethal in one animal whereas it resulted in a mild, non-lethal infection in a second animal [30]. The inconsistency in achieving infection as well as the resultant disease severity makes assessing the impact of supportive care on length of survival in mucosa-challenged animals very difficult and would require a large number of NHPs to detect. We therefore chose to use the accepted gold standard of targeting 1000 PFU intramuscular infection, recognizing that the disease it induces may differ from mucosal infection.

## Conclusion

In this highly lethal NHP Ebola virus model, we found that the animals developed progressive cardiovascular instability leading to profound and irreversible shock. The provision of advanced organ-supporting care (fluid administration, vasoactive medication, blood transfusion, hydrocortisone, and continuous bedside care) resulted in limited and time-dependent effects on the underlying EVD pathophysiology. These findings stress the need for further studies exploring the underlying cause(s) for progression to supportive care unresponsiveness; further development of animal models that replicate the full range of human EVD, rather than the fulminant course in the present use; and optimal use of vasoactive medications, and support ongoing development of EBOV-specific therapies.

## Additional files

Additional file 1: **Table S1.** Time to death in rhesus macaques infected with EBOV variant Makona C07 while cared for in a caged setting. (DOCX 13 kb)

Additional file 2: **Figure S1.** Vitals Signs over Time for the Uninfected Animal. Trends in vital signs over time for the uninfected animal. Heart rate (in beats per minute) and systolic/diastolic blood pressure (in mmHg) are plotted on the left Y-axis. Temperature is plotted on the right Y-axis. (TIF 575 kb)

## Data Availability

The datasets used and/or analyzed during the current study are available from the corresponding author on reasonable request.

## Abbreviations

BP: Blood pressure
BSL4: Biosafety level 4
EVD: Ebola virus disease
HPI: Hours post-infection
HR: Heart rate
IV: Intravenous
MAP: Mean arterial pressure
NHP: Non-human primate
TCID50: Tissue culture infectious dose (50%)
VAP: Ventilator-associated pneumonia
VIS: Vasoactive-inotropic score
