# Correction to: Impact of intensive care unit supportive care on the physiology of Ebola virus disease in a universally lethal non-human primate model

**DOI:** 10.1186/s40635-019-0283-9

**Published:** 2019-12-04

**Authors:** Guillaume Poliquin, Duane Funk, Shane Jones, Kaylie Tran, Charlene Ranadheera, Mable Hagan, Kevin Tierney, Allen Grolla, Amrinder Dhaliwal, Alexander Bello, Anders Leung, Cory Nakamura, Darwyn Kobasa, Darryl Falzarano, Lauren Garnett, Hugues Fausther Bovendo, Heinz Feldmann, Murray Kesselman, Gregory Hansen, Jason Gren, George Risi, Mia Biondi, Todd Mortimer, Trina Racine, Yvon Deschambault, Sam Aminian, Jocelyn Edmonds, Ray Saurette, Mark Allan, Lauren Rondeau, Sharron Hadder, Christy Press, Christine DeGraff, Stephanie Kucas, Bradley W. M. Cook, B. J. Hancock, Anand Kumar, Reeni Soni, Daryl Schantz, Jarrid McKitrick, Bryce Warner, Bryan D. Griffin, Xiangguo Qiu, Gary P. Kobinger, Dave Safronetz, Derek Stein, Todd Cutts, James Kenny, Geoff Soule, Robert Kozak, Steven Theriault, Liam Menec, Robert Vendramelli, Sean Higgins, Logan Banadyga, Guodong Liu, Md Niaz Rahim, Samantha Kasloff, Angela Sloan, Shihua He, Nikesh Tailor, Alixandra Albietz, Brad Pickering, Gary Wong, Michael Gray, James E. Strong

**Affiliations:** 1 National Microbiology Laboratory, Public Health Agency of Canada, 1015 rue Arlington Street, Winnipeg, Manitoba R3E 3R2 Canada; 2 Department of Pediatrics & Child Health, College of Medicine, Faculty of Health Sciences, University of Manitoba, Winnipeg, Manitoba Canada; 3 Department of Infectious Diseases and Medical Microbiology, College of Medicine, Faculty of Health Sciences, University of Manitoba, Winnipeg, Manitoba Canada; 4 Department of Anaesthesia and Medicine, College of Medicine, Faculty of Health Sciences, University of Manitoba, Winnipeg, Manitoba Canada; 5 Medtronic Canada, Winnipeg, Manitoba Canada; 6 National Centre for Foreign Animal Disease, Canadian Food Inspection Agency, Winnipeg, Manitoba Canada; 7 Vaccine and Infectious Disease Organization-International Vaccine Centre, University of Saskatchewan, Saskatoon, Canada; 8 Centre de Recherche en Infectiologie, Centre Hospitalier Universitaire de Québec, Université Laval, Québec, Canada; 9 Laboratory of Virology, Division of Intramural Research, National Institute of Allergy and Infectious Diseases, National Institutes of Health, Hamilton, USA; 10 Faculty of Critical Care, Royal University Hospital, Saskatoon, Saskatchewan Canada; 11 Infectious Disease Specialists, P.C., Missoula, MT USA; 12 Arthur Labatt Family School of Nursing, Western University, London, Ontario Canada; 13 Child & Women’s Health Programme, Winnipeg Regional Health Authority, Winnipeg, Manitoba Canada; 14 Cytophage Technologies, Inc, St. Boniface Hospital, Albrechtsen Research Centre, Winnipeg, Manitoba Canada; 15 Department of Surgery, Division of Pediatric Surgery, College of Medicine, Faculty of Health Sciences, University of Manitoba, Winnipeg, Manitoba Canada; 16 Regional Pharmacy, Winnipeg Regional Health Authority, Winnipeg, Manitoba Canada; 17 Department of Laboratory Medicine & Pathobiology, University of Toronto, Toronto, Ontario Canada

**Correction to: Intensive Care Med Exp (2019) 7:54**

**https://doi.org/10.1186/s40635-019-0268-8**

Please note that four authors (Logan Banadyga, Alixandra Albietz, Brad Pickering, and Gary Wong) have been erroneously omitted from the author list in the published original article [1].

Furthermore, note that three author names have been misspelled: ‘Niaz Md Rahim’ should be ‘Md Niaz Rahim’; ‘Ray Sourette’ should be ‘Ray Saurette’; ‘Darryl Schantz’ should be ‘Daryl Schantz’.

Please find the corrected author list in this article, with the importance of the contributions of the omitted authors reflected in the revised order of the list.

The authors apologize for any inconvenience caused.
