# Supplementary material for: Impact of intensive care unit supportive care on the physiology of Ebola virus disease in a universally lethal non-human primate model
Source: Intensive Care Med Exp. 2019 Sep 13;7:54. doi: 10.1186/s40635-019-0268-8 (PMC6744539; doi:10.1186/s40635-019-0268-8)
Supplement: Supplementary file 1 — Table S1. Time to death in rhesus macaques infected with EBOV variant Makona C07 while cared for in a caged setting. (DOCX 13 kb) [file 40635_2019_268_MOESM1_ESM.docx]

| **Animal #** | **Planned dose/route** | **Back titration dose** | **Time to death (d)** | **References** |
| --- | --- | --- | --- | --- |
| 1 | 1000 TCID50/ IM | 734 | 6 | [15, 17] |
| 2 | ‘’’’ | 734 | 8 | [15, 17] |
| 3 | ‘’’’ | 862 | 7 | [16, 17] |
| 4 | ‘’’’ | 862 | 8 | [16, 17] |
| 5 | ‘’’’ | 862 | 7 | [17] |
| 6 | ‘’’’ | 862 | 7 | [17] |

References

1. Brannan JM, He S, Howell KA, Prugar LI, Zhu W, Vu H, et al. Post-exposure immunotherapy for two ebolaviruses and Marburg virus in nonhuman primates. Nature Communications. 2019;10(1):105.
2. Wang H, Wong G, Zhu W, He S, Zhao Y, Yan F, et al. Equine-Origin Immunoglobulin Fragments Protect Nonhuman Primates from Ebola Virus Disease. Journal of Virology. 2018;93(5):e01548-18,
3. Banadyga L, Siragam V, Zhu W, He S, Cheng K, Qiu X. The Cytokine Response Profile of Ebola Virus Disease in a Large Cohort of Rhesus Macaques Treated With Monoclonal Antibodies. Open Forum Infectious Diseases 2019;6(3).
