# Supplementary figures and images for: Impact of intensive care unit supportive care on the physiology of Ebola virus disease in a universally lethal non-human primate model
Source: Intensive Care Med Exp. 2019 Sep 13;7:54. doi: 10.1186/s40635-019-0268-8 (PMC6744539; doi:10.1186/s40635-019-0268-8)

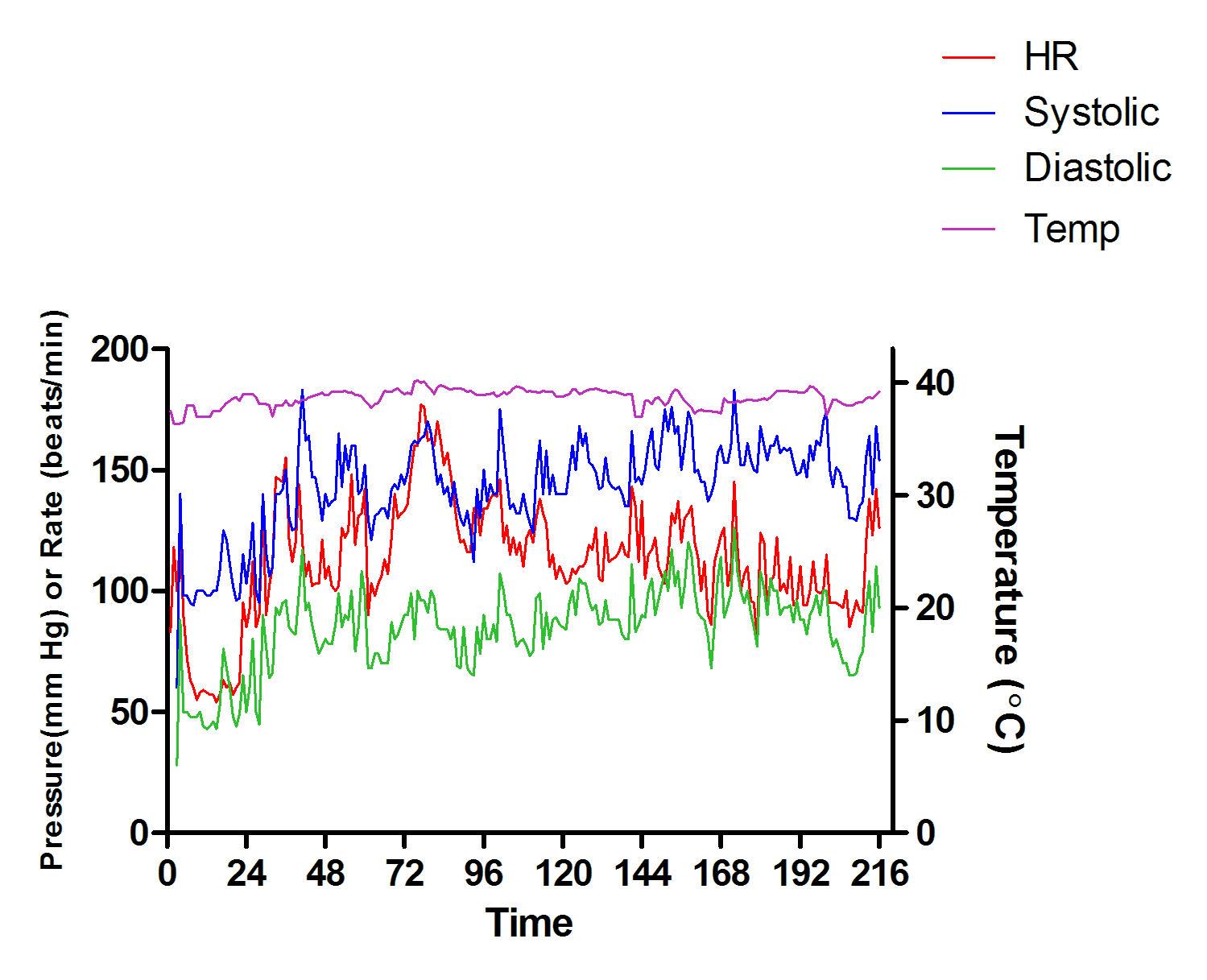

Supplement: Supplementary file 2 — Figure S1. Vitals Signs over Time for the Uninfected Animal. Trends in vital signs over time for the uninfected animal. Heart rate (in beats per minute) and systolic/diastolic blood pressure (in mmHg) are plotted on the left Y-axis. Temperature is plotted on the right Y-axis. (TIF 575 kb) [file 40635_2019_268_MOESM2_ESM.tif]
